# Assessment of Physico-Chemical and Toxicological Properties of Commercial 2D Boron Nitride Nanopowder and Nanoplatelets

**DOI:** 10.3390/ijms22020567

**Published:** 2021-01-08

**Authors:** Brixhilda Domi, Kapil Bhorkar, Carlos Rumbo, Labrini Sygellou, Spyros N. Yannopoulos, Rocio Barros, Roberto Quesada, Juan Antonio Tamayo-Ramos

**Affiliations:** 1 International Research Centre in Critical Raw Materials (ICCRAM), Universidad de Burgos, Plaza Misael Banuelos s/n, 09001 Burgos, Spain; bdomi@ubu.es (B.D.); crumbo@ubu.es (C.R.); rbarros@ubu.es (R.B.); 2 Foundation for Research and Technology Hellas-Institute of Chemical Engineering Sciences (FORTH/ICE-HT), P.O. Box 1414, GR-26504 Rio-Patras, Greece; kapilbhorkar@gmail.com (K.B.); sygellou@iceht.forth.gr (L.S.); sny@iceht.forth.gr (S.N.Y.); 3 CNRS, ISCR-UMR 6226, University of Rennes, F-35000 Rennes, France; 4 Departamento de Química, Facultad de Ciencias, Universidad de Burgos, 09001 Burgos, Spain; rquesada@ubu.es

**Keywords:** 2D boron nitride, eukaryotic model, nanotoxicity, cell viability, oxidative stress

## Abstract

Boron nitride (BN) nanomaterials have been increasingly explored for potential applications in chemistry and biology fields (e.g., biomedical, pharmaceutical, and energy industries) due to their unique physico-chemical properties. However, their safe utilization requires a profound knowledge on their potential toxicological and environmental impact. To date, BN nanoparticles have been considered to have a high biocompatibility degree, but in some cases, contradictory results on their potential toxicity have been reported. Therefore, in the present study, we assessed two commercial 2D BN samples, namely BN-nanopowder (BN-PW) and BN-nanoplatelet (BN-PL), with the objective to identify whether distinct physico-chemical features may have an influence on the biological responses of exposed cellular models. Morphological, structural, and composition analyses showed that the most remarkable difference between both commercial samples was the diameter of their disk-like shape, which was of 200–300 nm for BN-PL and 100–150 nm for BN-PW. Their potential toxicity was investigated using adenocarcinomic human alveolar basal epithelial cells (A549 cells) and the unicellular fungus *Saccharomyces* *cerevisiae*, as human and environmental eukaryotic models respectively, employing in vitro assays. In both cases, cellular viability assays and reactive oxygen species (ROS) determinations where performed. The impact of the selected nanomaterials in the viability of both unicellular models was very low, with only a slight reduction of *S. cerevisiae* colony forming units being observed after a long exposure period (24 h) to high concentrations (800 mg/L) of both nanomaterials. Similarly, BN-PW and BN-PL showed a low capacity to induce the formation of reactive oxygen species in the studied conditions. Even at the highest concentration and exposure times, no major cytotoxicity indicators were observed in human cells and yeast. The results obtained in the present study provide novel insights into the safety of 2D BN nanomaterials, indicating no significant differences in the toxicological potential of similar commercial products with a distinct lateral size, which showed to be safe products in the concentrations and exposure conditions tested.

## 1. Introduction

In recent years, the progress of nanotechnology has fueled the design and manufacturing of novel engineered nanomaterials (ENMs). In particular, different types of nanomaterials have been investigated for a wide range of potential applications [1,2,3,4]. Amongst them, low dimensional boron nitride (BN) materials, have attracted the attention of the scientific community due to their promising properties, such as high thermal conductivity, wide optical bandgap, strong ultraviolet emission, great mechanical stiffness, thermal stability, and chemical inertness [5,6,7]. BN consists of an equal number of boron and nitrogen atoms, which are arranged differently depending on pressure and temperature conditions, giving rise to distinct crystalline forms (hexagonal, rhombohedral, diamond-like cubic, and wurtzite). BN has been widely used in industrial applications for insulation, lubrication, fabrication of electronic parts, microwave-transparent objects, etc. [8,9]. The material is generally considered safe, being extensively used as well in the cosmetics industry, although not in its nanomaterial form [10,11]. More recently, BN-based nanomaterials have also raised the attention of the scientific community for their possible use in pharmaceutical and medical applications, such as cosmetics, drug delivery, scaffold materials for tissue engineering, and regenerative medicine, etc. [12]. As a consequence of the growth in the variety of applications of these nanomaterials, the increase of environmental and human exposure will be also higher, and may occur through multiple different pathways, which makes it necessary to study their potential toxicological effects. The availability of data on environmentally relevant concentrations (ERCs) of ENMs is mostly the result of estimations through exposure modeling [13]. So far, specific values for environmentally relevant concentrations of boron nitride nanoparticles have not been made available, while predicted ERCs for other frequently used ENMs in air, water, and soil, range from parts per trillion (ng/L or ng/kg) to parts per billion (μg/L or μg/kg) [14,15]. However, the toxicological testing of nanomaterials usually employs exposure conditions at higher concentrations (mg/L), which could be expected in exceptional scenarios (e.g., spills, uncontrolled waste discharges, etc.), and where toxicological responses are observable [16].

Several studies evaluating the toxicity of different BN nanoforms through in vitro, in vivo, and in silico approaches provide valuable hints on their safety [17,18,19,20,21]. However, the potential toxicity of distinct nanomaterial forms is still inadequately comprehended, with conflicting results reported in the scientific literature. Good biocompatibility has been reported for BN nanotubes, when exposed to HEK-293 human cells and freshwater planarians [21,22], while hollow BN nanospheres are able to induce apoptosis and inhibit the proliferation for both the androgen-sensitive LNCap and androgen-independent DU145 prostate cancer cells [23]. With regards to 2D BN nanomaterials, there is no clear consensus for their biocompatibility so far, but it seems to be dependent on cell type, dosage, and aspect ratio [12]. For instance, BN with a sheet-like structure produced adverse effects on human hepatoma HepG2 cells, decreasing cellular viability, enhancing intracellular ROS production, inducing adverse effects in mitochondrial depolarization, and membrane integrity has been recently reported [24]. Similarly, BN nanosheets changed from non-toxic to toxic towards SaOS2 cells when their diameters were reduced from the micro to nanometer range [25]. In a more recent study, in vivo and in vitro studies employing insect haemocytes, L929 mouse cells, and human erythrocytes showed that hexagonal BN (h-BN) nanosheets that functionalized with hydroxyl groups had low cytotoxicity, although the behavior of the insect immunocompetent cells was found to be altered [10].

The study of pristine and functionalized BN nanostructures as potential antimicrobial agents, through in vitro and in silico experimental approaches, have recently attracted the interest of researchers as well [17,26,27], aiming to develop polymer based biomedical devices protected against bacterial proliferation. 2D BN nanoparticles incorporated in polyhydroxybutyrate chitosan matrixes behaved as antibacterial agents against multi drug resistant *Escherichia coli* and *Staphylococcus aureus* strains, while showing good biocompatibility towards immortalized human keratinocytes (HaCaT) cell lines [28]. Similarly, BN flakes present in extruded low density polyethylene polymers displayed a bactericidal effect when evaluated against *E. coli, S. aureus*, *Staphylococcus epidermidis,* and *Pseudomonas aeruginosa*, strains [29]. Experimental and theoretical approaches employing transmission electron microscopy and molecular dynamics simulations suggest that the hydrophobicity of BN nanosheets can play a relevant role in damaging both bacterial outer and inner membranes [17]. In this regard, the ability of BN nanosheets to exert an antifungal effect is less known. A recent work that investigated the activity of h-BN nanoparticles against different bacterial species and *Candida* sp. M25 reported a low minimum inhibitory concentration and antibiofilm capacity towards the yeast strain [30]. However, BN spherical nanoparticles films composed by nanosheets and nanoneedles did not show an antifungal capacity against spores of distinct *Neurospora crassa* strains [27]. The little information available on the antimicrobial properties of BN nanomaterials is also an indicator of the limited knowledge that has been generated in relation to their potential environmental impact. Therefore, the objectives of the present work are (i) to generate new knowledge on the potential toxicity (e.g., as a potential inhalation target) and biocompatibility of 2D BN nanomaterials in humans, by using an in vitro model of widespread use such adenocarcinomic human alveolar basal epithelial cells (A549) [31], and (ii) to understand the environmental hazard of the selected nanomaterials towards the well-established ecotoxicological model *Saccharomyces cerevisiae* [32], providing as well novel insights into the potential antifungal properties of BN nanoforms.

## 2. Results and Discussion

### 2.1. Selection and Characterization of Commercial Boron Nitride

**Figure 1:**
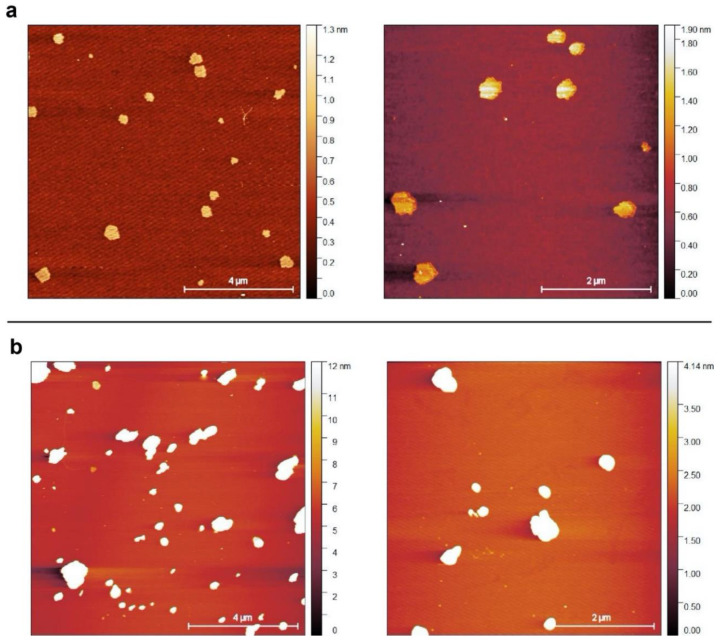
AFM images of BN nanopowder (**a**) and BN nanoplatelets (**b**). BN samples dispersions with a concentration of 20 mg/L were deposited by drop casting on a mica surface.

**Figure 2:**
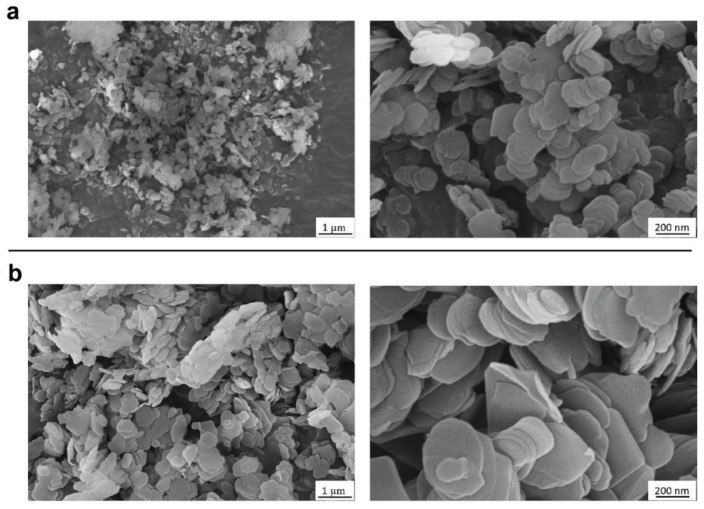
FE-SEM images of BN nanopowder (**a**) and BN nanoplatelets (**b**). Minute quantities of both samples were directly dropped on carbon tape.

**Figure 3:**
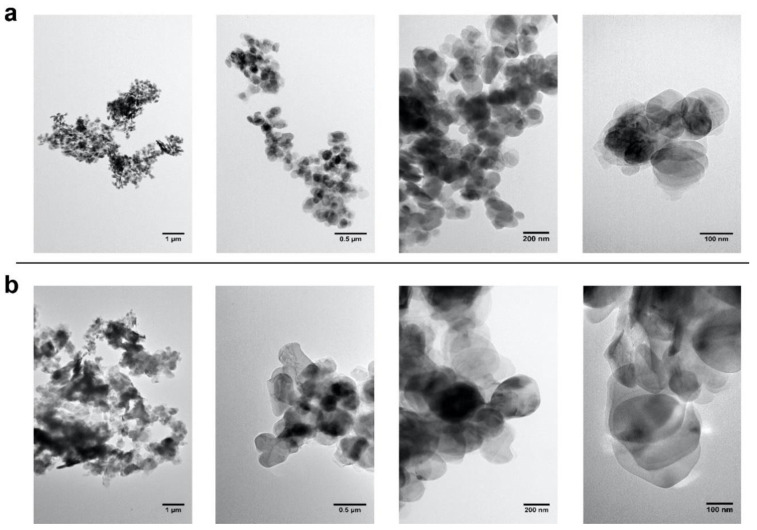
TEM images of BN nanopowder (**a**) and BN nanoplatelets (**b**). BN dispersions with a concentration of 20 mg/L were deposited by drop casting on carbon-coated copper grids.

In the present study, commercial BN nanopowder (BN-PW) and nanoplatelets (BN-PL) supplied by Sigma-Aldrich^®^ (Merck KGaA, Darmstadt, Germany) were selected. The characterization information provided by the supplier indicates an average particle size of <150 nm for BN-PW and a lateral size dimension of <1 micron for BN-PL. Many methodologies have been used by different authors to characterize the physico-chemical properties of nanomaterials, such as composition (e.g., ICP-MS, XPS, etc.), surface chemistry and atomic structure (XPS, Raman, etc.), charge (z-potential), size (SEM, TEM, dynamic light scattering, etc.), and morphology (AFM, SEM, TEM, etc.), aiming to understand their potential hazard when interacting with biological systems. To confirm the provider information and to further analyze the physico-chemical properties of the selected nanomaterials, a number of determinations using several of these methodologies were employed in the present study, and the obtained results have been described in Section 2.1 and Section 2.2 of this manuscript. To obtain insights into the products form and size, they were subjected to atomic force microscopy (AFM), field-emission scanning electron microscopy (FE-SEM), and transmission electron microscopy (TEM) analyses. As can be observed in Figure 1, AFM analyses confirmed the presence of particles populations and aggregates with a round shape, and a diameter in the nanoscale range for both BN products. Representative FE-SEM images (Figure 2) of the platelets and powders at two different magnifications revealed that the nanoparticles in both materials have a comparable disk-like geometry, while TEM images (Figure 3) confirmed that BN-PW and BN-PL have a 2D platelet-like shape. The main difference among the two samples at the morphological level was related to the diameter of the disk-like particles, which was observed to be of the order of 200–300 nm for BN-PL and 100–150 nm for BN-PW.

Additional insights into the diameter of the nanomaterials in different aqueous suspensions (water, human cells nanomaterials exposure media (DMEM 1% FCS), and *S. cerevisiae* cells culture media (YPD)) were obtained by determining their hydrodynamic size through dynamic light scattering (DLS). Both nanomaterials showed comparable average sizes (<500 nm) in all solvents tested, which indicates a low nanoparticle aggregation when resuspended in aqueous solutions with a significant concentration of soluble solutes, such as DMEM 1% FCS and YPD. The size distribution of the nanomaterials determined in the different aqueous suspensions is available as Supplementary Material (Figure S1).

Another relevant characteristic of nanoparticles when present in aqueous suspensions is their colloidal stability. The zeta potential is a key indicator of the stability of colloidal dispersions, so it was determined for BN-PW and BN-PL. As expected, both nanomaterials showed a better stability in water suspensions than when resuspended in human cells culture media and yeast cells culture media, which correlated well with the charges respectively determined for them in water (−35.3 mV ± 0.5 and −32.1 mV ± 0.5), DMEM 1% FCS (−10.0 mV ± 0.9 and −9.3 mV ± 0.6), and YPD (−14.7 mV ± 1.3 and −13.4 mV ± 1.7). The values obtained were similar to those previously reported for 2D BN and other bidimensional nanomaterials [33].

### 2.2. XPS and RAMAN Analysis

**Figure 4:**
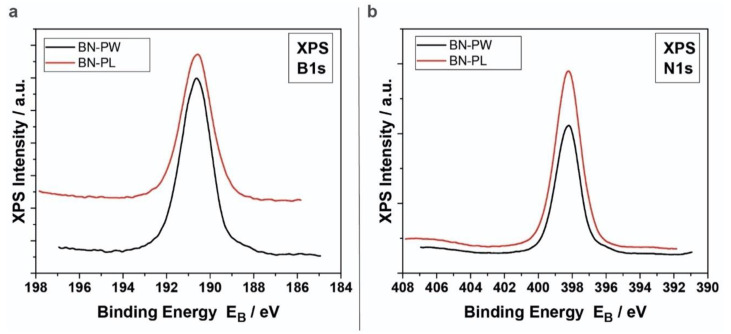
B1s (**a**) and N1s (**b**) XP spectra of BN nanopowder (BN-PW) and BN platetets (BN-PL) samples.

To analyze the surface chemistry (stoichiometry) and the atomic structure of the materials, XP and Raman spectra were collected and analyzed. Figure 4 shows the B1s and N1s XP spectra of BN-PW (black line) and BN-PL (red line) samples. For both samples, the B1s peak was found to be centered at 190.6 ± 0.1 eV, while the N1s peak was located at 398.1 ± 0.1 eV. These results are in concordance with the reported binding energies of hexagonal BN (h-BN) [34]. The atomic concentrations (%) of boron and nitrogen atoms in BN-PW (B: 51.00 ± 0.05; N: 49.00 ± 0.05) and BN-PL (B: 52.29 ± 0.06; N: 47.71 ± 0.06) corresponded as well to those expected for BN materials.

**Figure 5:**
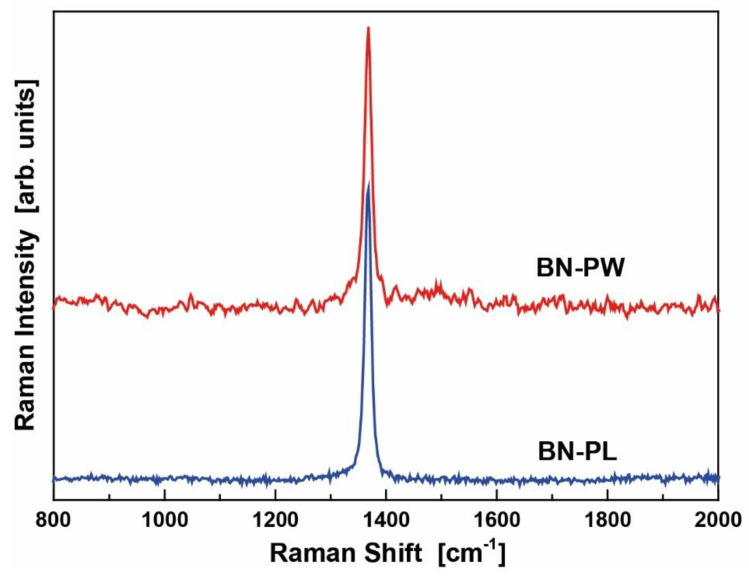
Raman spectra of BN powder and platelets.

The Raman spectra shown in Figure 5 also demonstrated the existence of the h-BN crystal phase. Fitting the spectra with Lorentzian lines revealed that the E_2g_ mode was located at 1367.5 and 1367.7 cm^−1^ for the BN-PL (blue line) and BN-PW (red line), respectively. Their half width at half maximum were 12.7 (BN-PL) and 14.7 cm^−1^ (BN-PW). Based on a correlation by Nemanich et al. [35], among the crystallite sizes and the Raman shift and width of the E_2g_ mode (the mode frequency shifted to higher energies and the width increased as the crystallite size decreased), the bandwidths of both BN samples indicated a slightly smaller crystallite size for BN-PW, which was in concordance to the observations in the performed microscopy analyses. Regarding the thickness dependence of the Raman spectra, it was first studied by Gorbachev et al. [36] who reported that the E_2g_ band of atomically thin BN flakes on Si/SiO_2_ substrates shifts with thickness. The BN monolayer exhibited a blue-shift of the order of 2–4 cm^−1^, whereas for more layers a red-shift was observed by 1–2 cm^−1^, in relation to the E_2g_ energy of the bulk h-BN. Contrasting results were reported by Li et al. [37], who found that the mono- and few-layer h-BN mechanically exfoliated flakes exhibit systematically a blue shift in the E_2g_ mode in comparison to the bulk energy. More recently, Cai et al. [38] found that in the absence of interactions with the substrate, mono- and few-layer BN flakes show no measurable shift in relation to the compound bulk form, suggesting that the observed Raman shift in related studies arises from the strain induced by the substrate. Based on these findings, and considering that the BN particles employed in the present study are not free standing, it can be concluded that the observed shift in BN-PW and BN-PL might be indicative of three-layers particles.

### 2.3. Toxicology Assessment Using Adenocarcinoma A549 Human Cells

**Figure 6:**
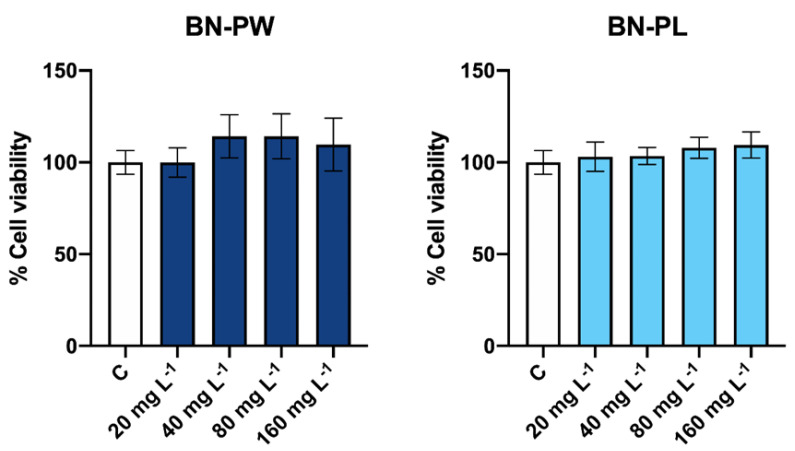
Human lung carcinoma (A549) cells viability after exposure to different concentrations of BN-PW and BN-PL. “C” corresponds to non-exposed cells. Two independent experiments, employing three technical replicates per exposure condition tested, were performed. One-way ANOVA followed by the Dunnett *post hoc* test was done to compare every mean with that of the control, and considered significant at *p* ≤ 0.05.

To assess the potential cytotoxic effects of commercial BN nanoforms, the human lung carcinoma cell line (A549) was selected as a cellular model, to study possible adverse effects in human health via the inhalation exposure of nanoparticles [39]. To determine the percentage of living cells after the BN exposure, the Neutral Red assay (one of the viability tests most widely used in nanotoxicological studies) was performed [40]. Therefore, cells were exposed to different concentrations (20, 40, 80, 160 mg/L) of both commercial BN-PW and BN-PL samples for a period of 24 h. As shown in Figure 6, after the A549 cells exposure to BN-PW and BN-PL, the viability of the cells was not reduced in the presence of any of the concentrations tested, indicating the absence of cytotoxicity in the employed conditions. Although BN nanomaterials are generally considered highly biocompatible [12], recent reports suggest that the toxicity of 2D BN depends on the cell type exposed, dosage, and nanomaterial aspect ratio. For instance, Liu et al. observed that human hepatoma HepG2 cells viability was significantly reduced in the presence of 30 mg/L BN sheet-like structured nanoparticles [24], while BN nanosheets changed from non-toxic to toxic towards SaOS2 cells when their diameter was reduced from the micro to nanometer range [25]. In a more recent study, in vivo and in vitro studies employing insect haemocytes, L929 mouse cells, and human erythrocytes showed that h-BN nanosheets functionalized with hydroxyl groups had low cytotoxicity, although the behavior of the insect immunocompetent cells was found to be altered [10].

**Figure 7:**
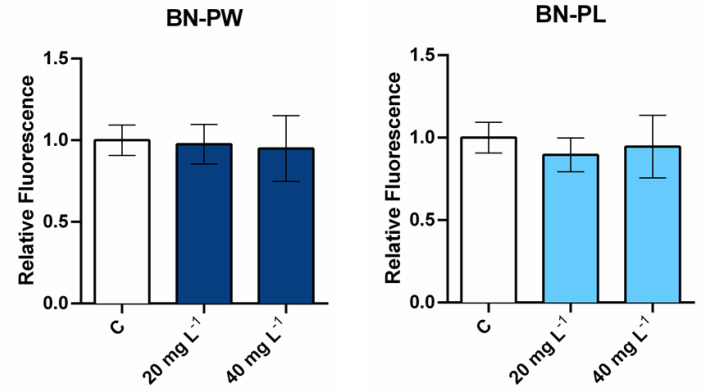
Determination of reactive oxygen species (ROS) levels in A549 cells exposed to 20 and 40 mg/L of BN-PW and BN-PL. “C” corresponds to non-exposed cells. Two independent experiments, employing three technical replicates per exposure condition tested, were performed. One-way ANOVA followed by the Dunnett *post hoc* test was done to compare every mean with that of the control, and considered significant at *p* ≤ 0.05. The figure including the positive control condition (H_2_O_2_ (20 μM)) is available as Supplementary Material (Figure S2).

Despite the absence of cell death after exposure to the selected BN samples, potential adverse effects on human cells following nanoparticles exposure could still occur due to the induction of oxidative stress [41]. It is well known that many different nanoparticle types can induce significant levels of reactive oxygen species (ROS), resulting in the cells’ inability to preserve normal physiological redox-regulated functions [42]. Due to their high oxidation potential, the overproduction of intracellular ROS can result in the damage of biomolecules and organelles, leading to necrosis, apoptosis, or even mutagenesis [42]. Therefore, to understand the possible presence of adverse effects at the sublethal level, we investigated the possible intracellular increase of ROS in cells exposed to the selected nanomaterials. As displayed in Figure 7, A549 cells were exposed to 20 and 40 mg/L of BN-PW and BN-PL for 1 h incubation, and the obtained results showed no ROS over production under the studied conditions.

Data available on oxidative stress induced by BN nanosheets in human cells is scarce, and the reported results are conflicting or difficult to compare. BN nanosheets, in the concentration range of 10–50 mg/L, did not induce ROS production in osteoblast cells exposed for 24 h [43]. However, in a more recent study, Mateti et al. reported a possible increase of ROS levels in osteoblast-like cells (SaOS2) exposed to BN nanosheets, although the nanomaterials concentration used was remarkably higher (1000 mg/L) [25]. Sheet-like BN nanoparticles also induced ROS formation in human hepatoma HepG2 cells exposed to relatively low concentrations (2–20 mg/L) [24]. Similarly, in a more recent study, DU145 and PNT1A prostate cells were exposed to 22 to 176 mg/L of BN nanoparticles, and an increase in ROS levels was observed in all cases [44].

### 2.4. Toxicology Assessment Using Saccharomyces Cerevisiae

**Figure 8:**
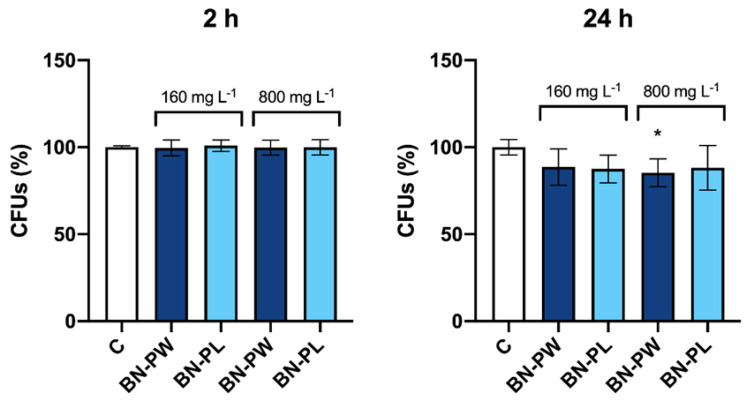
Determination of *S. cerevisiae* cells colony forming units (CFUs) after exposure to 160 and 800 mg/L of BN-PW and BN-PL, during 2 and 24 h. “C” corresponds to non-exposed cells. Two independent experiments, employing three technical replicates per exposure condition tested, were performed. One-way ANOVA followed by the Dunnett *post hoc* test was done to compare every mean with that of the control, and considered significant at *p* ≤ 0.05. * *p* ≤ 0.05.

The yeast *S. cerevisiae* is an extensively used eukaryotic model to comprehend fundamental molecular mechanisms and biological processes, which is also used as a tool for the toxicological evaluation of substances, such as pesticides, amongst other persistent and mobile chemicals, or ENMs [45,46]. Therefore, to evaluate the potential environmental impact of BN, yeast cells were exposed to two different concentrations of BN-PW and BN-PL (160 and 800 mg/L) at two exposure times (2 and 24 h), and subsequently their viability was assessed through colony forming units (CFUs) determination. As shown in Figure 8, only a small decrease on the cells’ viability was observed in the studied conditions after 24 h exposure. Although the antimicrobial properties of BN nanosheets has been previously explored, most of the studies performed have focused on bacteria, and data available on their antifungal potential is very low. A study performed by Kıvanç et al. reported a MIC value of h-BN nanoparticles against the yeast *Candida* sp. M25 of 3.25 mg/L [30]. This result contrasts with our observations, as *S. cerevisiae* cells proliferation was only slightly reduced, even in the presence of 800 mg/L of the nanomaterial. Further comparative analyses, employing the same exposure conditions and viability assays would be necessary to understand whether the differences observed between the studies are related to specificities from both yeast species, such as a distinct cell wall composition. In a recent study, pristine BN films composed by spherical nanoparticles formed by nanosheets and nanoneedles did not show an antifungal capacity against *Neurospora crassa* spores from different strains [27]. More studies are necessary to clarify the potential toxicity of BN nanomaterials against different fungal species.

**Figure 9:**
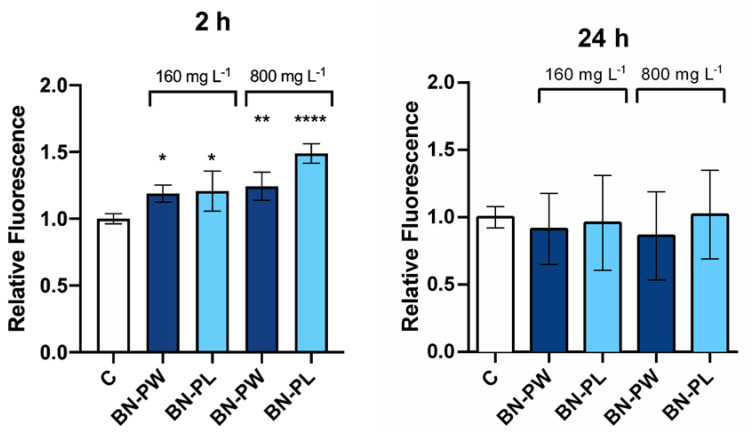
Oxidative stress determination of *S. cerevisiae* cells exposed to 160 and 800 mg/L of BN-PW and BN-PL during 2 and 24 h. The reported values are expressed in arbitrary units and correspond to the averages of two independent experiments (±standard deviation, SD), employing three technical replicates per exposure condition tested in each case. Differences were established using a one-way ANOVA followed by the Dunnett *post hoc* test to compare every mean with the control, and considered significant at *p* ≤ 0.05. * *p* ≤ 0.05, ** *p* ≤ 0.01, **** *p* ≤ 0.0001. The figure including the positive control condition (H_2_O_2_ (10 mM)) is available as Supplementary Material (Figure S3).

As mentioned above, the evaluation of the potential induction of ROS by ENMs is a standard toxicology assay that provides insights into possible cell damage, which can trigger cell death and apoptosis. The accumulation of ROS in yeast usually originates from internal metabolic processes connected to cell respiration, however, it can be similarly induced by environmental stress stimuli, such as nanoparticles exposure [47,48]. In yeast species, including *S. cerevisiae*, the consequences of ROS accumulation are programmed cell death, autophagy, necrosis, and upregulation of antioxidants mediated by complex transcriptional changes [49]. Therefore, to evaluate whether BN-PW and BN-PL were able to induce oxidative stress in *S. cerevisiae*, cells growing at the exponential phase were exposed to BN dispersion with a concentration of 160 and 800 mg/L for 2 and 24 h. As shown in Figure 9, the oxidative stress levels of yeast cells exposed for 2 h to BN-PW and BN-PL were slightly higher than those observed in the negative control. Specifically, the fluorescence signal increased 0.2 and 0.4 times more in the BN-PW and BN-PL samples present at the higher concentration (800 mg/L). However, ROS levels of yeast cells exposed to both nanomaterials for 24 h were not significantly different than those that could be observed in the negative control condition (non-exposed cells). The early ROS induction caused by the presence of BN-PW and BN-PL was lower than that induced by other 2D nanomaterials, such as graphene oxide and molybdenum disulphide, when yeast cells were exposed to them in comparable conditions [41,50].

## 3. Materials and Methods

### 3.1. Materials and Reagents

Chemicals employed were supplied by Sigma-Aldrich^®^ (Merck KGaA, Darmstadt, Germany) and Acros Organics (Thermo Fisher Scientific Inc., Madrid, Spain). BN nanopowder (BN-PW; ref 790532) and nanoplatelets (BN-PL; ref 900405) were purchased at Sigma-Aldrich^®^ (Merck KGaA, Darmstadt, Germany).

### 3.2. Atomic Force Microscopy

To perform and evaluate the AFM analysis, all the BN samples were dropped on a mica surface from aqueous suspensions. Images were recorded in tapping mode (set point of 500, 72 mV; drive amplitude 791.16; drive frequency 268.639), using silicon cantilevers AC160TS-R3 (aluminum reflex coating and tip radius <10 nm; Olympus, Japan), with a CYPHER ES instrument from Asylum Research (Oxford Instruments, Santa Barbara, California, USA). IGOR Pro 6.2 was used for data acquisition and control, while images analysis was done with ARgyle. The AFM analysis was performed at the laboratory of instrumental techniques unit service from the University of Valladolid.

### 3.3. Raman Analysis

The Raman analysis was done using an Ar ion laser (514.5 nm) in a focusing area of 2–3 μm. The scattered light was analyzed using a T-64000 (Jobin-Yvon, France) spectrometer (spectral resolution of ~2.0 cm^−1^). The Raman mode of Si single crystal at 520 cm^−1^ was used to calibrate the wavenumber scale of the spectra.

### 3.4. X-ray Photoelectron Spectroscopy

A SPECS Phoibos 100-1D-DLD and a non-monochromatized dual-anode Mg/Al X-ray source for XPS, contained within a UHV chamber (P ~ 5 × 10^−10^ mbar), were used to obtain XP spectra (MgKa at 1253.6 eV photon energy; analyzer pass energy 10 eV, FWHM of 0.85 eV for Ag3d_5/2_ line). The analyzed area was a rectangle of 4 × 20 mm^2^. The SpecsLab Prodigy software was used to collect and analyze the spectra. XPS peaks intensity, weighted with the corresponding RSF, were used to calculate atomic ratios.

### 3.5. Electron Microscopies

The high-resolution field-emission scanning electron microscope (FE-SEM) instrument (SUPRA 35VP, Zeiss, Germany) operating at 10 kV, and transmission electron microscope (TEM) analysis using a JEOL JEM-1011 high-resolution (HR) TEM coupled with a Gatan Erlangshen ES1000 W camera, were employed to investigate the texture and morphology of the commercially purchased BN nano-powders and nano-platelets were investigated. The TEM analysis was performed at the advanced microscopy unit service from the University of Valladolid.

### 3.6. Dynamic Light Scattering

Size determinations by dynamic light scattering (DLS) were done at Nanovex Biotechnologies S.L., employing the maximum nanoparticles concentrations used to expose human and yeast cells, using a Zetasizer ZS90 (Marlvern Instruments Ltd., UK).

### 3.7. Zeta-Potential Determination

Zeta-potential analyses were performed at Nanovex Biotechnologies S.L., by the mixed measurement mode phase analysis (M3-PALS) at room temperature, employing the maximum nanoparticles concentrations used to expose human and yeast cells, using a Zetasizer ZS90 (Marlvern Instruments Ltd., UK).

### 3.8. Assays in A549 Cells

The human alveolar carcinoma epithelial cell line A549 (ATCC, CCL-185) was cultured and maintained in a DMEM medium (Dulbecco’s Modified Eagle Medium), supplemented with 10% fetal calf serum (FCS) and 1% penicillin/streptomycin in a humidified incubator at 37 °C, in the presence of 5% CO_2_.

#### 3.8.1. A549 Cells Neutral Red Assay

Approximately 3 × 10^4^ cells per well were seeded in 96-well plates and exposed to 20, 40, 80, and 160 mg/L of the BN materials previously diluted in DMEM 1% FCS. After 24 h of incubation with nanomaterials, cells were washed with DPBS (Dulbecco’s Phosphate Buffered Saline) and the Neutral Red assay was performed as previously described [41]. Fluorescence measurements were recorded using a microplate reader (BioTek Synergy HT, Winooski, Vermont, USA), with an excitation wavelength of 530/25, and an emission wavelength of 645/40.

#### 3.8.2. A549 Cells ROS Determination

The DCFH-DA dye was used to quantify intracellular ROS levels. Seeded A549 cells in a 96-micro-well plate (around 3 × 10^4^ cells per well) were labelled, protected from the light, with 50 μM DCFH-DA in HBSS for 30 min. Subsequently, cells were washed with HBSS, and exposed for 1 h to the BN nanomaterials, previously diluted as well in HBSS (final concentrations: 20 and 40 mg/L). A BioTek Synergy HT (BioTek, Winooski, Vermont, USA) microplate reader was used to obtain fluorescence measurements (excitation wavelength, 530/25; emission wavelength, 645/40).

### 3.9. Yeast Culture

The *S. cerevisiae* BY4741 strain was grown at 30 °C, and maintained in a standard liquid and solid YPD medium (1% yeast extract, 1% yeast bacto-peptone, 2% glucose).

#### 3.9.1. Yeast Colony Forming Units (CFUs) Determination

*S. cerevisiae* cells were pre-grown on an YPD medium in an orbital shaker (30 °C and 185 rpm) until an OD 600 nm = 1 was reached, and then they were exposed to 160 or 800 mg/L of either BN-PL or BN-PW in the same medium culture, or cultured non exposed (negative control), in 24-well plates (final volume of 1 mL). Subsequently, culture samples were obtained after 2 and 24 h of exposure to the nanomaterials. To determine CFUs at both sampling times, 100 µL of cells were diluted 10^4^ times, in the case of 2 h exposure, and 10^5^ times, in the case of 24 h exposure, inoculated on a solid YPD medium (6% agar) plates, and incubated at 30 °C for 48 h.

#### 3.9.2. Yeast ROS Assay

The CM-H2DCFDA dye was used to quantify intracellular ROS levels in yeast, employing a protocol similar to that previously described by James et al. (2015) [51], recently adapted by our research group [41]. In short, cells were incubated with CM-H2DCFDA (7 μM) in DPBS at 30 °C and 185 rpm for 60 min, subsequently resuspended in an YPD liquid medium, and exposed to BN nanomaterials (160 and 800 mg/L) for 2 and 24 h. After two washing steps with DPBS, cells were incubated in AcLi 2M for 2 min, washed and incubated again for 2 min in a SDS (0.01%)-chloroform (0.4%) solution. Finally, the cells supernatant was transferred to a black opaque 96-micro-well plate, and fluorescence was measured with a Synergy-HT (BioTek, Winooski, Vermont, USA) microplate reader (excitation, 485; emission, 528).

### 3.10. Statistics

Statistical analysis data are presented as means ± SD. The one-way analysis of variance (ANOVA) was performed for multiple comparisons, followed by the Dunnet *post hoc* test. Statistical tests were carried out using Prism 6.0 (GraphPad Prism, GraphPad Software, Inc., San Diego, California, USA). *p*-values ≤ 0.05 were considered to indicate statistical significance.

## 4. Conclusions

The results obtained in the present work provide novel insights on the physico-chemical characteristics and the toxicological impact of commercial 2D BN nanomaterials on different eukaryotic models. The morphological analyses of commercial nanopowder and nanoplatelets determined a lateral size in the nanoscale range for both products, while the analyses of their structure and stoichiometry through Raman spectroscopy and XPS revealed characteristics in concordance with those of hexagonal BN (h-BN). The exposure analyses performed in human lung adenocarcinomic cells and the yeast *S. cerevisiae* indicated that both BN-PW and BN-PL have a low toxicological impact in the studied conditions. No reduction in cellular viability, nor oxidative stress production could be observed in exposed human cells, while minor effects were observed in exposed yeast cells even in the presence of very high nanomaterials concentrations. These results support the suitability of BN nanomaterials as 2D materials to develop future biomedical and environmental applications. However, since the degree of nanomaterial purity, size, shape, and thickness may have an influence on the biological response of different organisms, further studies are still necessary to increase the knowledge on the toxicological potential of other commercial nanoforms distinct to those studied in the present study, expanding as well the portfolio of model organisms tested, which will allow to enhance the knowledge on the potential hazard of BN nanomaterials against humans and the environment.

## Data Availability

The data presented in this study are available on request from the corresponding author.

## Supplementary Materials

The following are available online at https://www.mdpi.com/1422-0067/22/2/567/s1, Figure S1: Dynamic light scattering (DLS) analysis of BN-PW and BN-PL suspensions in water, DMEM 1% FCS, and YPD, Figure S2: ROS production of A549 cells treated with different concentrations of BN-PW and BN-PL, Figure S3: Oxidative stress (ROS) determination of *S. cerevisiae* cells exposed to 160 and 800 mg L^−1^ of BN-PW and BN-PL, during 2 h and 24 h.

## Abbreviations

AFM	Atomic force microscopy
BN	Boron nitride
DLS	Dynamic light scattering
TEM	Transition electron microscopy
FE-SEM	High-resolution field-emission scanning electron microscope
XPS	X-ray photoelectron spectroscopy
BN-PL	Boron nitride nanoplatelets
BN-PW	Boron nitride nanopowder
h-BN	Hexagonal boron nitride
SD	Standard deviation
DMSO	Dimethyl sulfoxide
DCFH-DA	2,7-dichlorofluorescin diacetate
ERC	Environmentally relevant concentration
ENM	Engineered nanomaterial
