# Supplementary material for: Assessment of Physico-Chemical and Toxicological Properties of Commercial 2D Boron Nitride Nanopowder and Nanoplatelets
Source: Int J Mol Sci. 2021 Jan 8;22(2):567. doi: 10.3390/ijms22020567 (PMC7827597; doi:10.3390/ijms22020567)
Supplement: Supplementary file 1 [file ijms-22-00567-s001.pdf]

Supplementary Materials

Assessment of physico-chemical and toxicological properties of commercial 2D boron nitride nanopowder and nanoplatelets

Domi B., ***et al.***

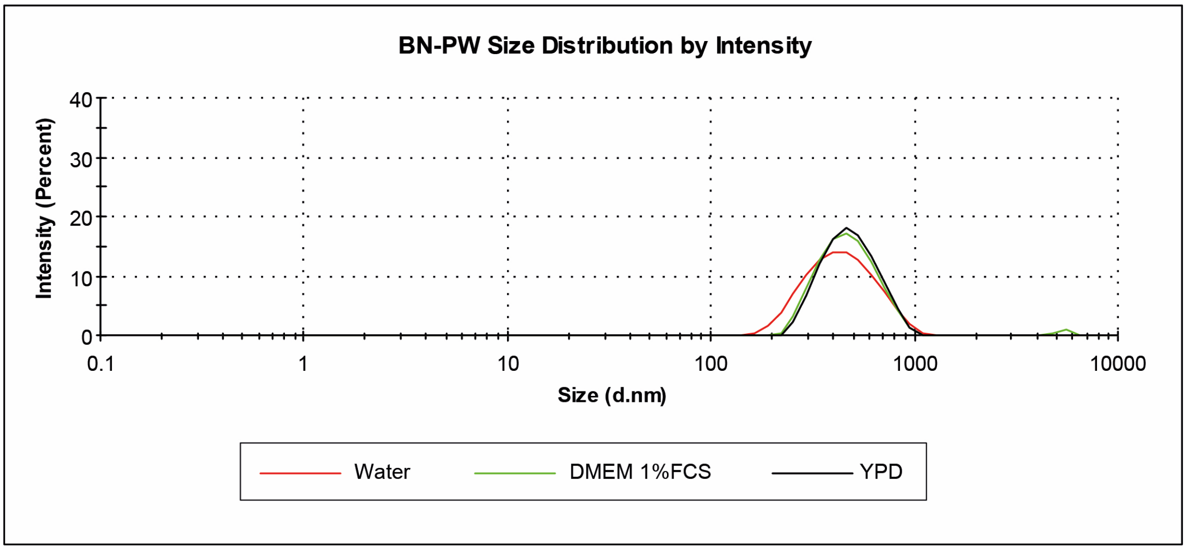

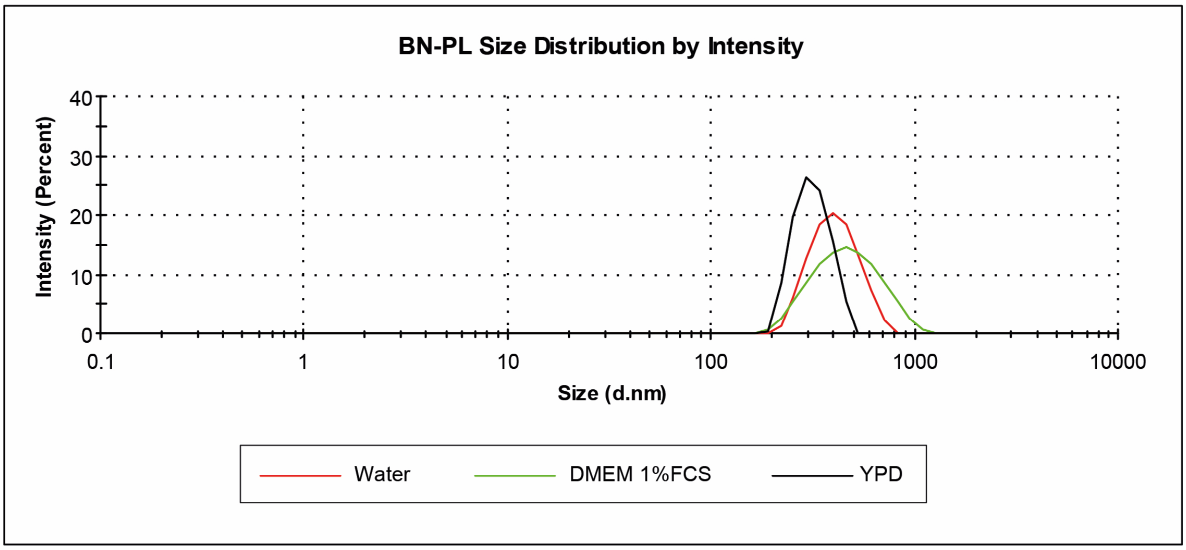

Figure S1. Dynamic light scattering (DLS) analysis of BN-PW and BN-PL suspensions in water, DMEM 1% FCS, and YPD.

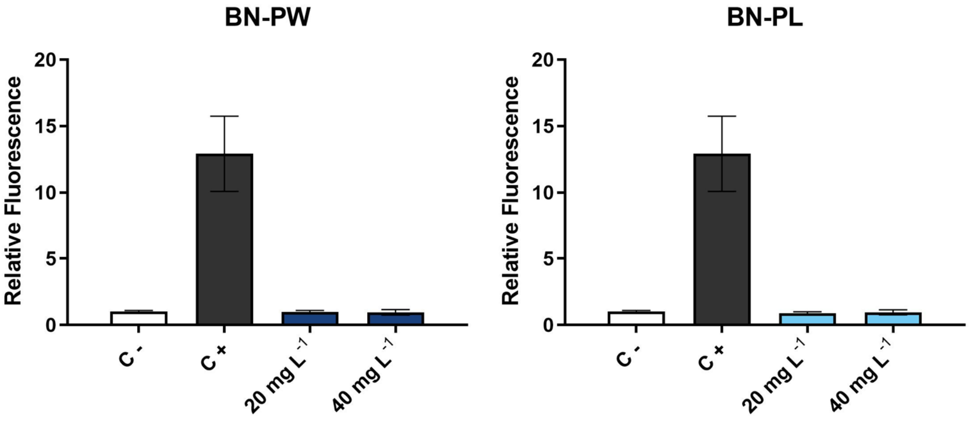

Figure S2. ROS production of A549 cells treated with different concentrations of BN-PW and BN-PL. A549 cells were exposed to H_2O_2 (20 µM) in the positive control condition. The reported values are expressed in arbitrary units and correspond to the averages of two independent experiments, employing 3 technical replicates per exposure condition tested in each case.

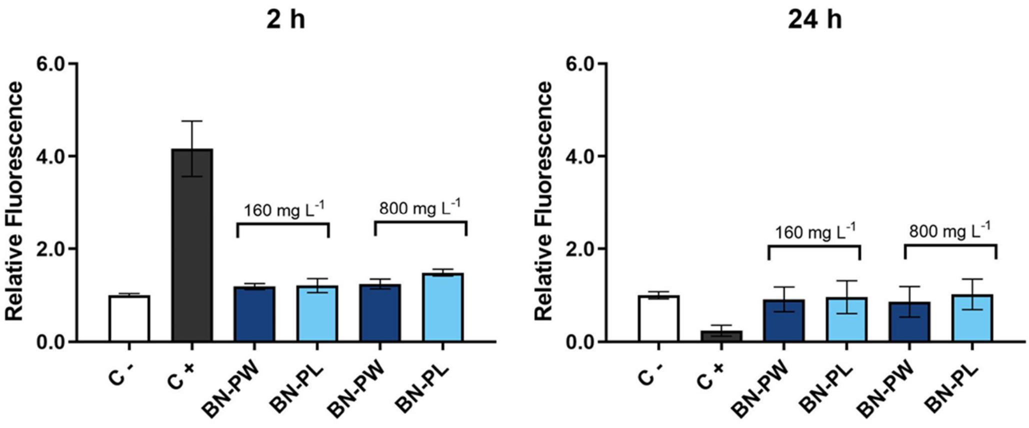

Figure S3. Oxidative stress (ROS) determination of *S. cerevisiae* cells exposed to 160 and 800 mg L^{-1} of BN-PW and BN-PL, during 2 hours and 24 hours. *S. cerevisiae* cells were exposed to H_2O_2 (10 mM) in the positive control condition. The reported values are expressed in arbitrary units and correspond to the averages of two independent experiments, employing 3 technical replicates per exposure condition tested in each case.
